# Neural Plasticity and Hearing‐Speech Development in Children with Auditory Brainstem Implants for Congenital Hearing Loss Due to Severe Inner Ear Malformation

**DOI:** 10.1002/advs.202406092

**Published:** 2025-03-28

**Authors:** Yu Zhang, Yongchuan Chai, Yiwen Zhang, Huan Jia, Zhaoyan Wang, Zhihua Zhang, Zhili Wang, Jie Chen, Xue Wang, Yun Li, Ying Chen, Hao Wu

**Affiliations:** ^1^ Department of Otolaryngology‐Head and Neck Surgery Shanghai Ninth People's Hospital Shanghai Jiaotong University School of Medicine No. 639, Zhizaoju Road Shanghai 200011 China; ^2^ Ear Institute Shanghai Jiaotong University School of Medicine No. 115, Jinzun Road Shanghai 200125 China; ^3^ Shanghai Key Laboratory of Translation Medicine on Ear and Nose Disease (14DZ2260300) No. 115, Jinzun Road Shanghai 200125 China

**Keywords:** auditory brainstem implant, electroencephalogram, functional near‐infrared spectroscopy, neural plasticity

## Abstract

Hearing and speech outcomes of pediatric patients with auditory brainstem implants (ABI) are variable, and the underlying developmental mechanism remains unexplored. This study aims to evaluate the effectiveness of ABI in pediatric patients with congenital non‐tumor hearing loss and elucidate the role of cortical plasticity in hearing and speech development. A prospective cohort study is conducted involving 112 consecutive pediatric patients who received ABI at a tertiary university‐based referral center from January 2019 to October 2023. The mean age at the time of surgery and postoperative follow‐up are 3.4±1.9 years and 25.7±11.7 months (3–48 months), respectively. The average percentage of elicited electrodes (eABR%) during the operation is 72.73±17.99%. Post‐activation, hearing and speech outcomes improved steadily. Younger age at implantation (<3 years), less severe inner‐ear malformation, and higher intraoperative eABR% (≥60%) are significantly associated with better hearing and speech outcomes. Mismatch negativity (MMN) responses are evoked in all children aged 12–18 months. Cortical functional connectivity developed after activation, particularly within the bilateral temporal/frontal lobes. Several factors contribute to hearing and speech development in children with ABI, and cortical plasticity plays a pivotal role. MMN amplitude and brain functional connectivity may serve as cortical indices for predicting long‐term outcomes.

## Graphical abstract

Data from a prospective cohort with 112 auditory brainstem implant users are analyzed. Younger age at implantation (<3 years), less severe inner‐ear malformation (common cavity, cochlear aplasia, and hypoplasia), and more intraoperative eABR evoked electrodes (≥60%) are associated with better hearing and speech outcomes. The amplitude of mismatch negativity and functional connectivity of left temporal with left frontal, right temporal with right frontal, and right temporal with left frontal cortices serve as pivotal cortical indices for predicting long‐term outcomes.

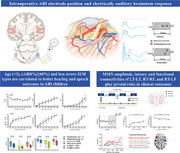

## 1 Introduction

Congenital hearing loss is the most common sensory defect at birth, with an incidence of 1‰–3‰,^[^ ^1^, ^2^ ^]^ and inner‐ear malformations (IEM) are present in ≈20% of children.^[^ ^3^, ^4^ ^]^ According to Sennaroglu,^[^ ^5^ ^]^ IEM can be classified as Michel deformity (MD), rudimentary otocyst (RO), cochlear aplasia (CA), cochlear hypoplasia (CH), common cavity (CC), cochlear incomplete partition, enlarged vestibular aqueduct, or cochlear nerve deficiency (CND). Most patients with severe IEM have bilateral severe‐to‐profound hearing loss and usually require early hearing implantation, including a cochlear implant (CI) and auditory brainstem implant (ABI). Regarding patients with severe IEM, CI is either impossible or less beneficial.^[^ ^6^ ^]^ ABI is a neuroprosthesis that could be used to bypass the auditory nerve and directly stimulate the cochlear nucleus to provide a sense of hearing.^[^ ^7^ ^]^

The first pediatric nontumor ABI was performed by Colletti in 2001.^[^ ^8^ ^]^ Since then, different centers have reported on their experiences of congenital hearing loss with IEM. These studies had limited sample sizes, and the individual outcomes of children with ABI were largely variable. Most participants acquired auditory perceptions. Some of these patients achieved open‐set speech recognition during long‐term follow‐up.^[^ ^9^, ^10^ ^]^ Only a few patients could converse via telephone within 3 years of implantation.^[^ ^11^, ^12^, ^13^ ^]^ Many factors can influence hearing and speech outcomes after ABI in children, such as associated disabilities, ABI device design, and electrode paddle placement.^[^ ^13^, ^14^, ^15^, ^16^ ^]^ Recent studies in children with hearing loss and CI have revealed that these outcomes are consequences of cortical plasticity.^[^ ^17^, ^18^, ^19^ ^]^ In children with severe IEM, the development of functional connectivity in the cerebral cortex is minimally influenced by sound, owing to little‐to‐no auditory information input. After ABI, the development of brain connectivity depends mainly on neural plasticity, which could be greatly influenced by the age at implantation, malformation severity, and electrode stimulation input.

In this study, based on a large series of children with ABI treated for congenital hearing loss at a tertiary referral center, we analyzed the long‐term effectiveness of implants and investigated the role of cortical plasticity in hearing and speech development for the first time.

## 2 Experimental Section

### 2.1 Patients

A total of 112 consecutive ABI surgeries in children with congenital hearing loss and severe IEM between January 2019 and October 2023 were included in this study. The diagnoses of bilateral profound sensorineural hearing loss and severe IEM were confirmed by audiological assessment and radiological imaging by the same experienced team. A total of 34 ears in 30 patients received CI with little‐to‐no benefit. Patients with syndromic hearing loss, neurological diseases, or mental disorders were excluded. The participants were implanted with unilateral ABI and followed up at 3, 6, 12, 18, 24, 36, and 48 months after the first fit.

The study was approved by the Ethics Committee of the Ninth People's Hospital affiliated with Shanghai Jiaotong University School of Medicine (SH9H‐2021‐T151‐1, ChiCTR2100048681). Informed consent was obtained from the parents or guardians of all enrolled patients.

### 2.2 ABI Surgery and Intraoperative Electrophysiological Assessment

All surgical procedures were performed by a senior surgeon. All children underwent ABI surgery using either the retrosigmoid or retrolabyrinthine approach. The ABI devices used in this study were the MEDEL Concerto in four cases, MEDEL Synchrony in 78 cases, and NUROTRON WH‐01A in 30 cases with 12 or 16 electrodes.

**Figure 1:**
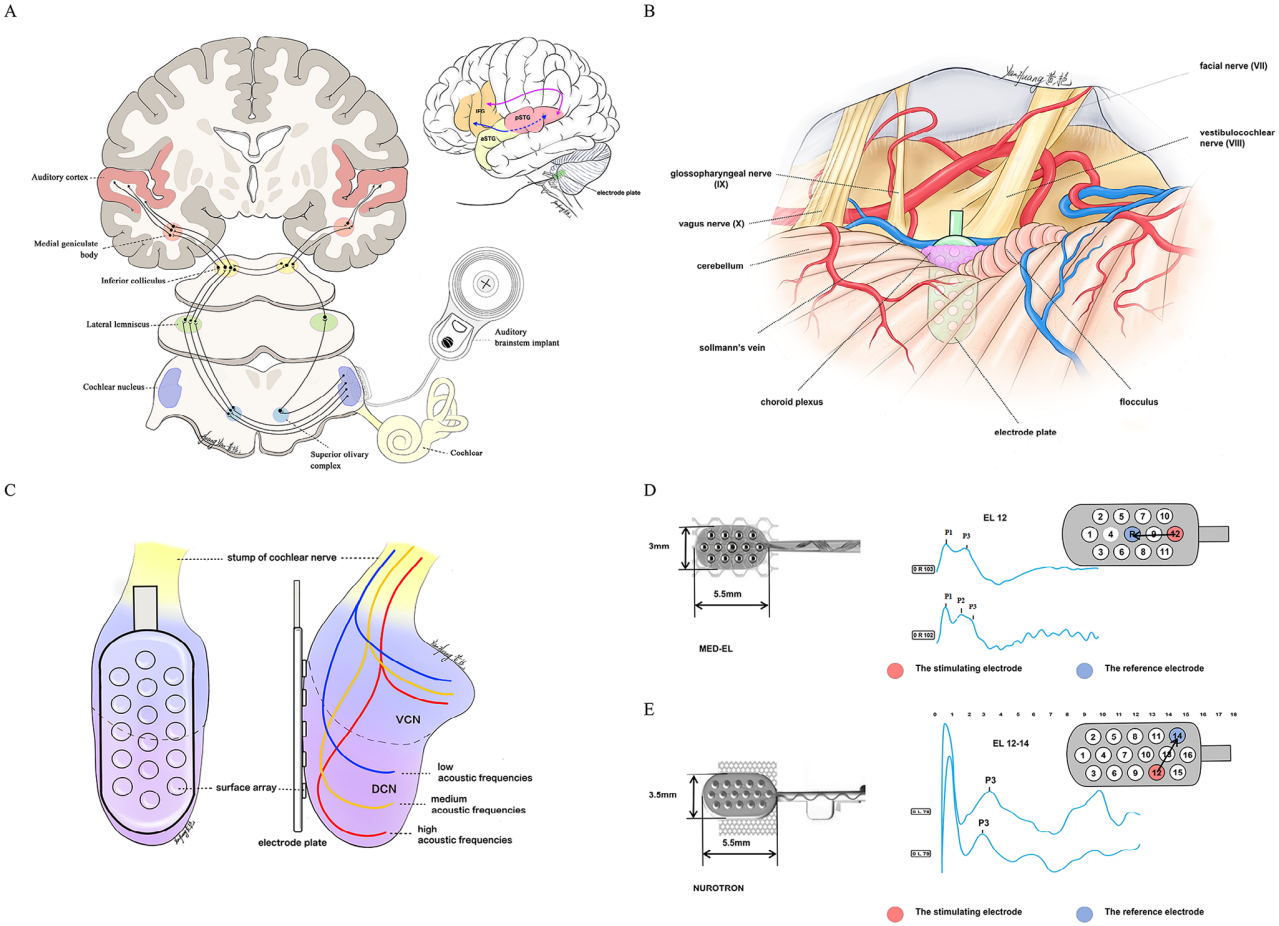
Location of the ABI paddle and the intraoperative eABR waveform. A) The auditory pathway of ABI patients. B) Location of the ABI electrode paddle in the lateral recess of the fourth ventricular lateral recess of the brainstem. C) Anatomical relationship between the cochlear nucleus and electrode paddle. D) Paddle of the MED‐EL ABI and its intraoperative eABR waveform: 12 electrodes plus one reference electrode, size = 5.5 * 3.0 mm. The red and blue electrodes represent the stimulation and reference electrodes, respectively. E) The paddle of NEUROTRON WH‐01A ABI and its intraoperative eABR waveform: 16 electrodes, size = 5.5 * 3.5 mm. This product uses an electrode adjacent to the stimulating electrode (red) as the reference electrode (blue).

The positioning of the ABI electrode paddle was guided by the anatomical structure and the fourth ventricular lateral recess of the brainstem and subsequently confirmed by intraoperative electrically evoked auditory brainstem responses (eABRs).^[^ ^20^, ^21^ ^]^ The eABRs were examined according to clinical standards and evaluated by experienced audiologists. The number of averages was dictated by the quality of the response and was typically set to 500–1000. The multipeak waveforms evoked in the first 2–3 ms of the electrical stimulus were considered the response of the auditory pathway from the cochlear nucleus to the inferior colliculus, and a long peak was regarded as a non‐auditory response.^[^ ^22^, ^23^ ^]^ Considering the anatomy of the cochlear nucleus and the size of the electrode paddle, an effective auditory response was often difficult to elicit for all electrodes; therefore, repositioning was often required, as shown in **Figure** **1**.

### 2.3 Activation, Fitting, and Programming

The ABIs were activated 6 weeks after surgery in an ambulatory operating room under sedation, with cardiac and respiratory monitoring by anesthetists. The eABR assessments were conducted again to determine the response of the electrode array. During implant activation, children were closely observed for non‐auditory responses, including body sway, ocular nystagmus, and facial tremors.^[^ ^24^, ^25^ ^]^ Electrodes that induced nonauditory effects were inactivated. Following the first fitting, the eABR assessment and programming were conducted at each follow‐up. Device programming was based on both eABR and behavioral measurement.^[^ ^24^ ^]^

### 2.4 Hearing and Speech Assessment

The first behavioral assessment was carried out 1 day after activation and then at each follow‐up. Behavioral assessments included the following: The aided pure tone average (PTA, dB) was calculated with mean thresholds of 0.5, 1, 2, and 4 kHz; categories of auditory perception (CAP) rating scale classified auditory ability using scores ranging from 0 to 9;^[^ ^26^ ^]^ the speech intelligibility rate (SIR) classified speech ability with scores ranging from 1 to 5.^[^ ^27^ ^]^ The scores of the Meaningful Auditory Integration Scale (IT‐MAIS/MAIS) and the Meaningful Use of Speech Scale (MUSS) ranged from 0–40.^[^ ^28^, ^29^ ^]^ The higher the scale score, the better the hearing and speech development.

### 2.5 Functional Neuroimages

Functional neuroimaging was performed at each follow‐up using multichannel electroencephalography (EEG, Brain Products) and a functional near‐infrared spectroscopy (fNIRS, NIRSout) system. A special test cap was used to simultaneously record the neural responses of electrical activity and changes in oxyhemoglobin concentration. The cap contained 32 fNIRS optodes and 13 EEG electrodes, guided by the International 10–20 system. The electrodes included F1, F2, FCz1, FCz2, Fz, FCz, Cz, CPz, Pz, POz, Oz, TP9, and TP10, and one electrode located at the tip of the nose was used as the reference electrode. The fNIRS optodes formed 46 fNIRS channels (source‐detector pairs) with an inter‐optode separation of 30 mm, covering the participants’ temporal lobes and part of their frontal lobes. The EEG and fNIRS signals were recorded at 1000 and 6.25 Hz sampling rates, respectively.

The mismatch negativity (MMN) paradigm was used to assess frequency discrimination. Trials were conducted in a quiet environment, and 70 dB pure tones were played in the sound field. Each trial contained 10 blocks, each of which included 25 stimuli, with a 20–30s interval between them. A 500 Hz standard and 1000 Hz deviant stimuli were presented in a pseudorandom sequence at proportions of 80% and 20%, respectively. The duration of each stimulus was 500 ms and the inter‐stimulus interval (ISI) was 300 ms. The stimuli were delivered through two loudspeakers located 100 cm from the participants using Eprime 2.0.

The EEGLAB toolbox was used to preprocess and analyze the EEG data.^[^ ^30^ ^]^ The recording window included −200 ms pre‐stimulus time to +600 ms post‐stimulus time. The recorded data were filtered from 1–30 Hz. ≈250 responses were obtained from each participant. The responses greater than ±20 µV were rejected offline and the remaining were averaged to compute a final grand‐averaged waveform for the individual patient.

NIRSLAB software was used to preprocess and analyze the fNIRS data.^[^ ^31^ ^]^ Channels with a coefficient of variance greater than 7.5% for either wavelength were excluded from the analysis. Subsequently, the fNIRS data were checked for motion artifacts through visual inspection and corrected accordingly. A low‐frequency cutoff (0.01 Hz) and a high‐frequency cutoff (0.2 Hz) were used with a 15% roll‐off width. Finally, absorption data were converted into concentration data using the modified Beer‐Lambert Law. The coherence was calculated for all channels for each participant.^[^ ^32^ ^]^

### 2.6 Statistical Analysis

Data were presented as percentages for categorical variables and mean (SD) for continuous variables. The Kolmogorov‐Smirnov test was used to examine the normality of continuous variables. Regarding the two‐group comparison of continuous variables, an independent‐sample t‐test was used for normally distributed data, whereas the Mann‐Whitney U test was used for other data. Concerning intragroup comparisons at two different time points, a paired‐sample t‐test was used. To evaluate hearing and speech outcomes, repeated‐measures analysis of variance was applied, with the change from baseline as the dependent variable and time as the independent variable. Subgroup analysis was applied according to eABR% (<60% or ≥60%), age (<3 or ≥3 years), and IEM type. To reduce the dimensionality of cortical indices, principal component analysis (PCA) was performed with the original variables of MMN amplitude, MMN latency, and functional connectivity. The original variables produced the first and second principal components (PC1 and PC2) with eigenvalues >1, and their contributions to behavioral outcomes, including CAP and IT‐MAIS/MAIS, were analyzed using the Mann‐Whitney U test.

Statistical significance was defined as a two‐tailed *p* value < 0.05. All statistical analyses were performed using IBM SPSS software (version 27.0).^[^ ^33^ ^]^

## 3 Results

The mean age at implantation was 3.4±1.9 years (ranging from 1.2 to 8.6). All patients were diagnosed with profound congenital hearing loss and bilateral air conduction ABR thresholds of > 95 dB nHL. The residual hearing was further analyzed in the less severe IEM group (n = 25). Regarding Auditory‐Steady State Responses (ASSR) results, a total of 10 patients (40%, 10/25) showed responses on at least one frequency, indicating that they had residual hearing. Thirty children underwent CI surgery before ABI, and their IEM types included 24 CND and 6 CH, as shown in **Table** **1**. The surgical procedures used were the retrosigmoid and retrolabyrinthine approach in 89 and 23 cases, respectively, with repositioning of the ABI electrodes occurring an average of 2.3 times in this series. Major complications were not observed. Cerebrospinal fluid leakage with electrode paddle translocation occurred in two cases, that were cured by revision surgery.

**Table 1:** Demographic characteristics of auditory brainstem implant users.

Patient	Age at ABI surgery			Total
	<3 years	3‐6 years	>6 years	
Total	48 (41.0%)	55 (50.1%)	9 (8.57%)	112
Sex				
Male	33 (28.6%)	29 (26.7%)	2 (1.90%)	74 (57.1%)
Female	15 (12.4%)	26 (23.8%)	7 (6.67%)	48 (42.9%)
IEM type				
MD	3 (2.68%)	6 (5.36%)	0	9 (8.04%)
RO	7 (6.25%)	6 (5.36%)	0	13 (11.6%)
CC	4 (3.57%)	2 (1.79%)	1 (0.89%)	7 (6.25%)
CA	24 (21.4%)	4 (3.57%)	7 (6.25%)	35 (31.3%)
CH	3 (2.68%)	6 (5.36%)	0	9 (8.04%)
CND	6 (5.36%)	30 (26.8%)	0	36 (32.1%)
CO	1 (0.89%)	1 (0.89%)	1 (0.89%)	3 (2.68%)
Implantation side				
Left	5 (4.46%)	15 (13.4%)	4 (3.57%)	24 (18.8%)
Right	43 (38.4%)	40 (35.7%)	5 (4.46%)	88 (78.6%)
CI before ABI				
Left	2(1.79%)	8(7.14%)	1(0.952%)	11(9.82%)
Right	1(0.952%)	13(11.61%)	1(0.952%)	15(13.39%)
Bilateral	1(0.952%)	3(2.86%)	0	4(3.57%)

IEM, inner‐ear malformation; MD, Michel deformity; RO, rudimentary otocyst; CC, common cavity; CA, cochlear aplasia; CH, cochlear hypoplasia; CND, cochlear nerve deficiency; CO, cochlear ossification.

### 3.1 Electrophysiological, Hearing, and Speech Outcomes

**Figure 2:**
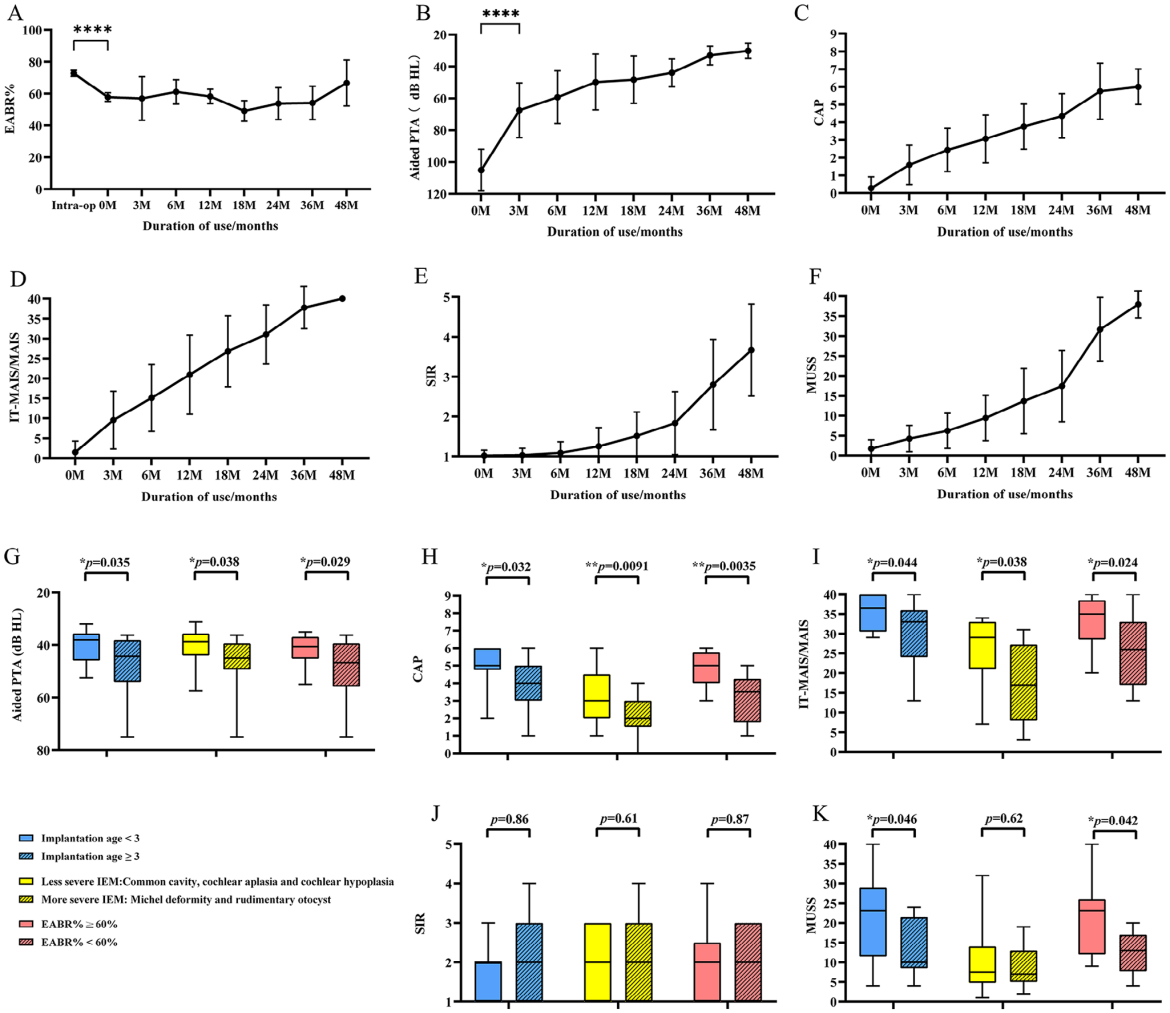
eABR%, hearing, and speech outcomes in ABI children over time and comparison of hearing and speech outcomes (24 months) between different subgroups according to implantation age, IEM type, and the percentage of intraoperative eABR evoked electrodes A) eABR% during surgery (n = 112), activation (0, n = 112), 3 (n = 112), 6 (n = 105), 12 (n = 96), 18 (n = 76), 24 (n = 45), 36 (n = 22), and 48 months (n = 4). There was a significant decrease in eABR% at activation compared with the intraoperative recordings (*p*<0.0001), which remained stable during follow‐up. B) The results of aided PTA showed marked improvement from 0 to 3 months (*p*<0.0001). C,D) The outcomes of hearing scales (CAP and IT‐MAIS/MAIS) showed continuous improvement after activation. E,F) The outcomes of the speech scales (SIR and MUSS) showed slow development during the first 24 months and then an obvious acceleration from 24 to 48 months. G–K) The blue, yellow, and red boxes represent ABI children implanted before the age of 3 years, with a less severe IEM, and an eABR% ≥60%, respectively. The blue, yellow, and red boxes with a shadow represent ABI children implanted after the age of 3 years, with a more severe IEM, and eABR% <60%, respectively. ABI children implanted before the age of 3 years achieved better outcomes in terms of aided PTA (*p* = 0.035), CAP (*p* = 0.032), IT‐MAIS/MAIS (*p* = 0.044), and MUSS (*p* = 0.046) at 24 months than those implanted after the age of 3 years. ABI children with less severe IEM achieved better hearing ability in terms of aided PTA (*p* = 0.038), CAP (*p* = 0.0091), and IT‐MAIS/MAIS (*p* = 0.038) than those with more severe IEM. Children with eABR% ≥60% achieved significantly better hearing and speech ability in terms of aided PTA (*p* = 0.029), CAP (*p* = 0.0035), IT‐MAIS (*p* = 0.024), and MUSS (*p* = 0.042) at 24 months compared to those from the other group.

All children underwent regular follow‐ups at 3 (n = 112), 6 (n = 105), 12 (n = 96), 18 (n = 76), 24 (n = 45), 36 (n = 22), and 48 months (n = 4). The results of electrophysiological assessments and hearing/speech outcomes at each follow‐up are summarized in **Figure** **2**, including the percentage of elicited electrodes (eABR%), aided PTA, CAP, IT‐MAIS/MAIS, SIR, and MUSS.

The eABR% values at each follow‐up visit are shown in Figure 2A. The eABR% was 72.73%±17.99% intraoperatively, and it decreased to 57.80%±23.35% at activation and then remained stable during subsequent follow‐ups (67.00%±25.76%) up to 48 months.

All users acquired hearing sensations and speech abilities after ABI activation. The hearing outcomes at each follow‐up, including aided PTA, CAP, and IT‐MAIS/MAIS, are summarized in Figure 2B–D. Hearing ability rapidly improved in the first three months of ABI use and then progressively ameliorated, lasting for at least 48 months. At 0, 3, 24, and 48 months, the mean PTA values were 105.01±13.07, 67.48±17.17, 43.92±8.74, and 30.10±4.73 dB HL; the mean CAP scores were 0.28±0.63, 1.60±1.12, 4.36±1.25, and 6.00±1.00; and the mean IT‐MAIS/MAIS scores were 1.47±2.79, 9.54±7.24, 31.04±7.40, and 40.00±0.00, respectively.

Speech developmental outcomes, including SIR and MUSS, are shown in Figure 2E,F. Unlike hearing outcomes, the development of SIR and MUSS was slower during the first 24 months than during later stages. At 0, 24, 36, and 48 months, the mean SIR scores were 1.02±0.14, 1.83±0.79, 2.80±1.14, and 3.67±1.16, respectively, and the mean MUSS scores were 1.71±2.25, 17.43±8.94, 31.67±8.03, and 38.00±3.46, respectively.

### 3.2 Factors Related to Hearing and Speech Outcomes

To explore potential factors associated with hearing and speech outcomes in patients, children were divided into groups based on age at implantation (≥3 years, n = 21 vs <3 years, n = 24), IEM type (less severe IEM [CC, CA, and CH], n = 25 vs more severe IEM [MD and RO], n = 20), and eABR% (≥60%, n = 26 vs <60%, n = 19). Subsequently, hearing and speech outcomes at 24 months in each subgroup were compared using the Mann‐Whitney U test, as shown in Figure 2G–K.

In the subgroup analysis regarding age at implantation, the repeated measure analysis of variance showed that the interaction group time was statistically significant in the aided PTA (*p* = 0.0028) and CAP (*p* = 0.011), but not in the IT‐MAIS (*p* = 0.18), MUSS (*p* = 0.28), and SIR (*p* = 0.25) scores. At 24 months, the mean aided PTA was 40.25±5.94 dB HL, CAP was 5.12±1.25, IT‐MAIS/MAIS was 35.70±4.57, and MUSS was 20.81±9.75 in the younger implantation age group (<3 years old) compared to 47.13±10.34, 4.05±1.19, 30.14±7.73, and 13.22±7.23 dB HL in the older implantation age group (≥3 years old), respectively (*p* = 0.035*, p* = 0.032*, p* = 0.044*, p* = 0.046).

In the subgroup analysis of IEM types, the patients in the less severe IEM group achieved better hearing development, as the mean aided PTA was 45.91±0.69 dB HL, CAP was 4.05±1.19, and IT‐MAIS/MAIS was 24.73±9.74. Conversely, in the more severe IEM group, the values were 47.11±10.62, 3.24±1.38, and 17.77±9.56 dB HL, respectively (*p* = 0.038*, p* = 0.0091*, p* = 0.038).

Concerning the subgroup analysis of the intraoperative eABR %, electrophysiological assessments were very important in determining the correct placement of the ABI device during surgery, and the intraoperative eABR% was also critical for hearing and speech development. We found that eABR% ≥60% intraoperatively was associated with better hearing and speech ability during follow‐up. The average aided PTA was 41.75±5.42 dB HL at 24 months. The mean CAP, IT‐MAIS/MAIS, and MUSS scores were 4.79±1.02, 33.12±6.10, and 20.79±8.84, respectively. Conversely, the children with eABR% <60% exhibited lower values: 48.98±11.91 dB HL for aided PTA, 3.17±1.47 for CAP, 26.14±9.17 for IT‐MAIS/MAIS, and 12.50±5.54 for MUSS (*p* = 0.029*, p* = 0.0035*, p* = 0.024*, p* = 0.042), respectively.

### 3.3 Cortical Plasticity Characterized by MMN and Functional Connectivity

**Figure 3:**
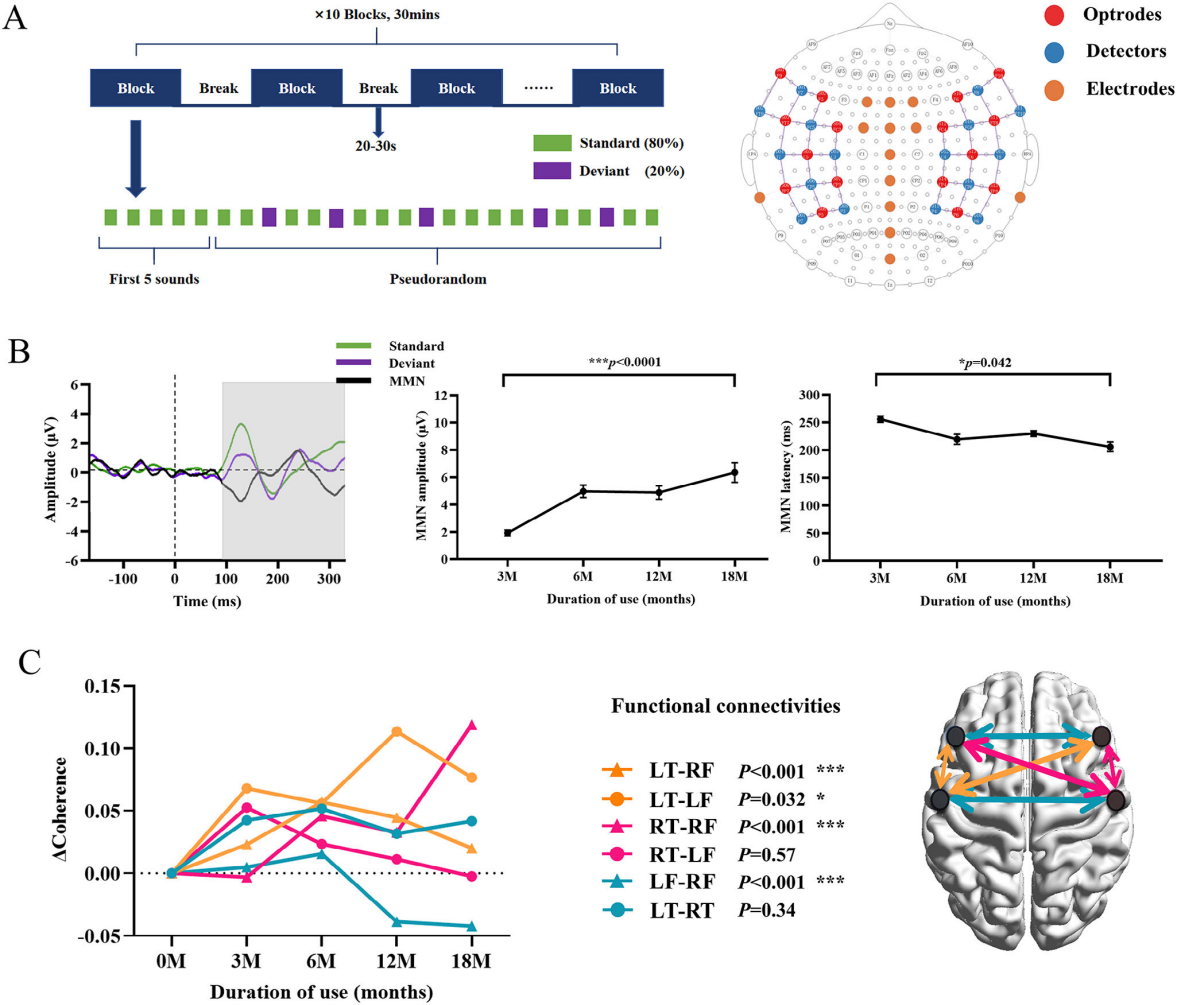
Developmental features of cortical plasticity characterized by MMN and functional connectivity in patients with ABI. A) Paradigm of the Study. B) The gray region shows a significantly different time (from 98 to 320 ms) between the standard and deviant conditions. The MMN amplitude significantly increased (*p*<0.0001) and the latency significantly shortened (*p* = 0.042) from 0 to 18 months. C) Functional connectivity of interest and changes in each functional connectivity are presented over time. There were significant improvements in connectivity in LT‐RF (*p<*0.001), LT‐LF (*p* = 0.032), and RT‐RF (*p*<0.001); LF‐RF decreased (*p*<0.001), and RT‐LF and LT‐RT showed no significant changes. LT‐LF, left temporal with left frontal; LT‐RF, left temporal with right frontal; RT‐RF, right temporal with right frontal; RT‐LF, right temporal with left frontal; LT‐RT, left and right frontal; LF‐RF, left and right temporal.

In 22 children with ABI, we assessed neural functional development using EEG and fNIRS from activation to 18 months at each follow‐up and performed repeated‐measures analysis of variance. The paradigm is shown in **Figure** **3**A.

First, we analyzed the MMN data of these children to reveal the function and maturation of the auditory‐related cortices. The average MMN at 18 months is shown in Figure 3B. There was a significant difference between the standard and deviant waveforms in the interval of 98–320 ms presenting in the gray region. MMN showed remarkable development from 3 to 18 months, with the amplitude significantly increasing from 1.92±1.17 µV to 6.35±3.39 µV (*p*<0.0001), and latency shortening from 255.80±38.54 ms to 206.00±35.93 ms (*p* = 0.042).

To further elucidate the process of cortical development, we analyzed functional connectivity between the temporal and frontal cortices, which are the most important cortices in hearing and speech processing. Functional connectivity of interest included left temporal with left frontal (LT‐LF), left temporal with right frontal (LT‐RF), right temporal with right frontal (RT‐RF), right temporal with left frontal (RT‐LF), left and right frontal (LF‐RF), and left and right temporal (LT‐RT), as shown in Figure 3C. The development of each connectivity varied. From activation to 18 months, significant improvements were observed in LT‐RF (*p*<0.0001), LT‐LF (*p* = 0.032), and RT‐RF (*p*<0.0001), whereas LF‐RF (*p*<0.0001) decreased notably. However, the functional connectivity in the RT‐LF and LT‐RT groups showed no significant changes.

### 3.4 Cortical‐Index‐Based PCA of Hearing Outcomes

**Figure 4:**
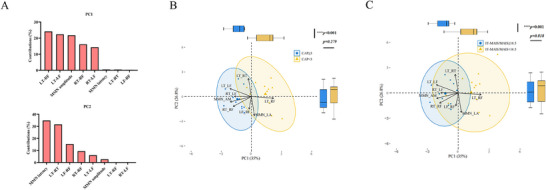
The cortical‐index‐based principal component analysis of hearing outcomes, including CAP and IT‐MAIS/MAIS at 18 months. The eight cortical indices included functional connectivity (LT‐RF, LT‐LF, RT‐RF, RT‐LF, LF‐RF, and LT‐RT) and MMN amplitude and latency. A) Box plots show the percentage of explained variance for PC1 and PC2. B,C) The contributions of the eight indices to PC1 and PC2 are indicated by arrows. Patients with ABI were divided into two groups based on the median CAP and IT‐MAIS/MAIS for comparison. The MMN amplitude and functional connectivity LT‐LF, RT‐RF, and RT‐LF made considerable contributions to PC1 in both CAP (*p*<0.001) and IT‐MAIS/MAIS (*p* = 0.001), whereas connectivity LT‐RF negatively affected them.

To understand the cortical mechanisms of hearing development after ABI, 2D PCA was performed to reveal the potential correlation between cortical indices and hearing outcomes, as shown in **Figure** **4**. The cortical indices included MMN amplitude/latency and functional connectivity including LT‐LF, LT‐RF, RT‐RF, RT‐LF, LF‐RF, and LT‐RT. After dimension reduction, three components were extracted. The first two principal components accounted for 61.8% of the variance in the data as PC1 (35.0%) and PC2 (26.8%). The LT‐LF (24.17%), LT‐RF (22.33%), and MMN amplitude (21.77%) mainly contributed to PC1, whereas RT‐RF (16.20%) and RT‐LF (14.36%) made considerable contributions to PC1. MMN latency (34.80%) and LT‐RT (31.55%) mainly contributed to PC2. LT‐RF (15.21%), RT‐RF (9.40%), and LT‐LF (6.09%) contributed less to PC2, as presented in Figure 4A.

To further distinguish the potential factors of various clinical behavioral outcomes, we performed the same subgroup analysis using PC1 and PC2 as shown in Figure 4B,C. In the subgroup of CAP <3 (median) and ≥3, there is a significant difference in PC1 between the two groups (*p*<0.0001). In the subgroup of IT‐MAIS/MAIS <16.5 (median) and ≥16.5, there is also a significant difference in PC1 between the two groups (*p* = 0.001). However, no significant differences in PC2 were observed between the two groups, regardless of whether they were divided by the median CAP (*p* = 0.279) or IT‐MAIS/MAIS (*p* = 0.818). These results indicate that the MMN amplitude and functional connectivity of LT‐LF, RT‐RF, and RT‐LF made considerable contributions to both CAP and IT‐MAIS/MAIS, while the connectivity of LT‐RF negatively affected them.

## 4 Discussion

The variable outcomes of pediatric ABI have been attributed to many factors such as device design, intraoperative electrode placement, and individual developmental state. In this study, we present the results of children who underwent ABI procedures for profound congenital non‐tumor hearing loss due to severe IEM and elucidate the role of cortical plasticity during hearing and speech development.

An important reason for the variation in ABI outcomes is intrinsic individual differences among these children. In previous studies on CI in children with congenital hearing loss, many factors were found to be related to hearing and speech outcomes including age at implantation, IEM, associated disability, electrode design, and intraoperative fine structure preservation.^[^ ^34^, ^35^, ^36^ ^]^ Among these factors, the age at implantation has been highlighted as a significant determinant of outcomes in children with CI. Previous studies on CI noted that hearing and speech abilities could reach a plateau at ≈24 months, while ABI needed more time.^[^ ^37^, ^38^ ^]^ In this study, children with ABI usually showed an accelerated period of speech development after 24 months and took more time to reach the plateau, generally at least 48 months. Several studies have reported the age of 3 years as a critical age to undergo CI surgery to achieve auditory and language development close to normal levels.^[^ ^39^, ^40^ ^]^ In this study, we also concluded that ABI surgery at an age younger than 3 years was significantly correlated with better hearing and speech outcomes. Similarly, our study showed that 3 years was a critical age for patients with ABI. Our previous study on a cohort of children with CI and normal cochlea aged 6–12 months demonstrated an overall developmental benefit.^[^ ^41^ ^]^ However, the possibility of ABI surgery in children younger than 12 months has not been investigated. Many studies on CI have demonstrated a critical period for central auditory and spoken language development.^[^ ^42^ ^]^ If no sound input is fed into the auditory system during this sensitive period, the auditory cortex could be reorganized using other sensory inputs. Therefore, early implantation is necessary to enable the normal development of the auditory pathways in children with congenital deafness. Although the minimum age at which our team performed surgery was 11 months, we usually recommend implantation at an age above 12 months because the surgical risks are lower and cranial structures are larger.

The type of IEM is also an important factor influencing ABI outcomes. IEM is the result of cochlear developmental arrest at different embryonic stages and includes MD, RO, CA, CC, CH, and incomplete cochlear partition with or without CND (cochlear nerve aplasia [CNA] or cochlear nerve hypoplasia [CNH]).^[^ ^43^ ^]^ ABI is an absolute indication in more severe IEM (MD, RO, CA, and CNA) and a relative indication in less severe IEM (CC, CH, ICP, and CNH) in which a CI was first implanted. We subdivided the children with ABI into two groups based on the severity of IEM. Subgroup analysis revealed worse hearing and speech development in the more severe IEM group. In our previous study using magnetic resonance imaging with diffusion tensor imaging in children with congenital hearing loss, children with IEM showed more fiber impairment in the hearing and language pathways, which might be the mechanism behind the worse outcomes.^[^ ^44^ ^]^

Surgical factors also impact post‐implantation hearing and speech outcomes. A critical step during surgery is the precise placement of the electrode array on the surface of the cochlear nucleus, which is located using anatomical landmarks and electrophysiological assessments.^[^ ^20^, ^21^ ^]^ EABR is a valid tool for measuring the cortical auditory response during CI and ABI surgery.^[^ ^45^ ^]^ EABR in the malformed cochlea usually has a longer latency than that in the normal cochlea with CI.^[^ ^46^ ^]^ Meanwhile, in ABI, eABR is evoked by direct electrical stimulation of the cochlear nucleus,^[^ ^47^ ^]^ and the resulting multi‐peak responses in the first 2–3 ms represent the activation of the auditory brainstem pathway.^[^ ^48^ ^]^ Previous studies^[^ ^49^, ^50^ ^]^ have suggested that the intraoperative eABR of an electrode usually indicates auditory perception at the electrode postoperatively in both patients with neurofibromatosis II and children with congenital hearing loss. In addition, more active electrodes in mapping were associated with better long‐term perceptual outcomes. Although these studies further emphasize the usefulness of attempting to ensure definitive eABR responses when inserting an ABI into the cochlear nucleus, it remains unclear whether eABR could be used as a predictor of postoperative outcomes. Other intraoperative electrophysiological parameters such as local evoked potentials (LEPs), which are related to the activity of stimulated neurons in the vicinity of the area between the array and cochlear nucleus, have also been used to predict hearing outcomes. In a previous study where surgery was performed in 16 patients with ABI, LEP measurement could predict auditory perception and was correlated with both behavioral thresholds and the most comfortable behavioral levels. However, LEP measurement has a high false positive rate, and its threshold does not definitively correlate with eABR, suggesting that it does not necessarily distinguish auditory perception from non‐auditory responses.^[^ ^51^ ^]^ In the present study, the intraoperative percentage of eABR‐evoked electrodes was analyzed, which could define the maturation of the cochlear nucleus. The results demonstrated that eABR% was significantly correlated with postoperative outcomes. If the eABR% exceeded 60% in surgery, the children could achieve a CAP of 6 or better in 24 months and attain open‐set speech recognition. eABR% has the potential to predict hearing/speech development and support fitting in children with ABI.

Neural plasticity is the ability to alter brain anatomy and connectivity, as well as the capacity for structural and functional reorganization, which is maximal in neonates and infants.^[^ ^52^ ^]^ In children with congenital hearing loss, the absence of early hearing experience triggers adaptive organization in the auditory system, leading to increased excitability of neurons toward other inputs, such as visual and somatosensory inputs.^[^ ^53^ ^]^ After auditory implantation, the hearing system undergoes another form of reorganization to adapt to new electrical sound inputs. Previous studies have shown that adaptive changes during hearing deprivation significantly affect hearing and speech outcomes, requiring time and effort to reorganize these changes after cochlear implantation.^[^ ^54^, ^55^ ^]^ The MMN response reflects the maturation of the auditory pathway through sound discrimination ability, which is characterized by increasing amplitude and shortened latency. In normal‐hearing infants, MMN demonstrates increased sensitivity to subtle auditory differences during the first year of life.^[^ ^56^, ^57^ ^]^ Previous studies have also summarized the developmental pattern of the auditory system in children with CIs, where shorter latency and larger amplitude of MMN remained consistent with more proficient speech perception.^[^ ^58^, ^59^ ^]^ A notable period of MMN maturation in children with CIs occurs 3–6 months after CI placement.^[^ ^60^ ^]^ Here, we present the first study on cortical plasticity in children with ABI and profound congenital hearing loss. In this study, children with ABI required significantly more time to adapt and reorganize, as indicated by the EEG and fNIRS analyses. The MMN amplitude increased and the latency shortened during the period from activation to 18 months in children with ABI, whose development lagged behind that of normal‐hearing infants and children with CIs. Delayed maturation of the auditory cortex in children with ABI results in slower speech acquisition.

The typical brain functional connectivity involved in hearing and speech processes after hearing restoration also develops in specific patterns. Functional connectivity in infants exhibits dynamic changes that vary across different brain regions, involving both strengthening and pruning, particularly within 3 months after birth.^[^ ^61^, ^62^, ^63^ ^]^ Specifically, functional connectivity between the left temporal and inferior frontal gyri (IFG) increases significantly and is closely related to speech perception.^[^ ^64^ ^]^ The left IFG has been identified as a cortical region potentially involved in effortful listening.^[^ ^65^ ^]^ An increase in functional connectivity between the left temporal and left frontal cortices was found in children with ABI, which might be associated with increased listening effort due to the need for more cognitive resources to effectively discriminate speech signals. This is similar to what was observed in children with normal hearing under a low signal‐to‐noise ratio and children with CI.^[^ ^66^ ^]^ Bilateral temporal connectivity is important for effective processing of auditory signals. In a previous study with preterm infants, bilateral temporal connectivity increased rapidly after birth owing to earlier auditory exposure.^[^ ^67^ ^]^ In children with ABI, we also measured bilateral temporal connectivity but failed to observe an increase during follow‐up, possibly due to inadequate and indistinct sound inputs. Additionally, connectivity in the right hemisphere was much lower than that in the left hemisphere in children with ABIs, which is consistent with normal‐hearing infants and shows a left‐dominant function in language.^[^ ^68^, ^69^ ^]^

We used PCA to analyze the contributions of eight cortical indices (MMN [amplitude and latency] and functional connectivity [LT‐LF, LT‐RF, RT‐RF, RT‐LF, LF‐RF, and LT‐RT]) to hearing outcomes (IT‐MAIS/MAIS and CAP). The results showed that MMN amplitude and functional connectivity LT‐LF and RT‐RF had positive effects, whereas LT‐RF had a negative effect on hearing development in children with ABI. MMN amplitude and latency reflect the response strength and speed of neurons related to sound input, respectively.^[^ ^53^ ^]^ In this study, the amplitude significantly contributed to hearing outcomes, but the latency did not. This finding possibly indicates that children with ABI have increased neural activity to adapt to sound input. However, the latency did not show a shortening trend as in normal‐hearing children, and neurons might require a longer response time for recognition and discrimination. In previous studies, the functional connectivity LT‐LF was identified as an important marker of auditory and speech abilities in individuals with normal hearing.^[^ ^70^ ^]^ Our findings demonstrated that the functional connectivity of RT‐RF and LT‐LR played a significant role in hearing development in children with ABI. Increased recruitment in the right hemisphere provides enhanced neural processing to compensate for inadequate sound input and immature hearing. In normal infants, the functional connectivity of frontal cortices decreases from birth to 3 months of age and increases from 3 to 6 months of age (“U”‐shaped change) due to neural pruning to process sound input more efficiently and specifically.^[^ ^62^ ^]^ The U‐shaped pattern may represent a reorganization of connectivity between the frontal and other cortical regions in normal‐hearing infants.^[^ ^71^, ^72^ ^]^ In this study, the interhemispheric connectivity of LT‐RF gradually increased and negatively affected hearing development in children with ABI until 18 months after implantation. This suggests that the connectivity remained immature and required additional auditory experience to establish their effectiveness. It remains unknown whether the functional connectivity LT‐RF in children with ABI shows a similar “U”‐shaped developmental pattern as in normal infants.

In the present study, we analyzed the role of neural plasticity in hearing and speech development in children with ABI using EEG and fNIRS. Understanding the mechanisms of cortical plasticity in children with ABI is crucial for guiding rehabilitation strategies and identifying potential targets for neural regulation and needs further exploration.

### 4.1 Strengths and Limitations

The strengths of the present study are as follows: (1) it has a large sample of pediatric patients with ABI and a long follow‐up time of up to 48 months; (2) it uses simultaneous cortical assessments by EEG and fNIRS and reveals critical cortical indicators related to hearing and speech outcomes after ABI; and (3) it examines the possible cortical mechanisms of variable hearing outcomes in pediatric ABI.

We acknowledge the following limitations of this study. (1) The longest follow‐up time was 48 months, and cortical assessment was carried out until 18 months after activation in 22 patients. As previously reported, ABI recipients require more time to achieve stable gains in behavioral outcomes, especially speech ability, which is estimated to last 4–5 years.^[^ ^10^ ^]^ Therefore, we will continue the follow‐up process. (2) The MMN paradigm includes only frequency discrimination and lacks more complicated speech tasks such as tone, vowel, and sentence recognition. These more specific paradigms will be considered in future studies, which seem very challenging for children with ABI with only 18 months of use. (3) To avoid repetitive use of sedatives, cortical assessments were performed without sedation. Individual factors such as movement, attention, and mood might have influenced the results. However, all patients with an ABI were assessed in a cooperative state by the same experienced audiologist to avoid bias.

## Conflict of Interest

The authors declare no conflict of interest.

## Author Contributions

Yu Zhang, Yongchuan Chai, and Yiwen Zhang contributed equally to this work. Hao Wu and Ying Chen have full access to all data in the study and take responsibility for the integrity of the data and the accuracy of the data analysis. Hao Wu, Yu Zhang, and Yun Li designed this study. Hao Wu, Yongchuan Chai, Huan Jia, and Zhihua Zhang completed the surgery. Yun Li, Yu Zhang, and Yiwen Zhang completed the assessment regarding the operation and follow‐up. Yu Zhang and Jie Chen carried out the statistical analyses. Hao Wu, Yu Zhang, and Ying Chen interpreted the results and drafted the manuscript. All authors critically reviewed the manuscript.

## Data Availability

The data that support the findings of this study are available from the corresponding author upon reasonable request.
